# Towards a Video Passive Content Fingerprinting Method for Partial-Copy Detection Robust against Non-Simulated Attacks

**DOI:** 10.1371/journal.pone.0166047

**Published:** 2016-11-18

**Authors:** Zobeida Jezabel Guzman-Zavaleta, Claudia Feregrino-Uribe

**Affiliations:** Computer Science Department, Instituto Nacional de Astrofísica, Óptica y Electrónica (INAOE), Sta. Ma. Tonanzintla, Puebla, México; West Virginia University, UNITED STATES

## Abstract

Passive content fingerprinting is widely used for video content identification and monitoring. However, many challenges remain unsolved especially for partial-copies detection. The main challenge is to find the right balance between the computational cost of fingerprint extraction and fingerprint dimension, without compromising detection performance against various attacks (robustness). Fast video detection performance is desirable in several modern applications, for instance, in those where video detection involves the use of large video databases or in applications requiring real-time video detection of partial copies, a process whose difficulty increases when videos suffer severe transformations. In this context, conventional fingerprinting methods are not fully suitable to cope with the attacks and transformations mentioned before, either because the robustness of these methods is not enough or because their execution time is very high, where the time bottleneck is commonly found in the fingerprint extraction and matching operations. Motivated by these issues, in this work we propose a content fingerprinting method based on the extraction of a set of independent binary global and local fingerprints. Although these features are robust against common video transformations, their combination is more discriminant against severe video transformations such as signal processing attacks, geometric transformations and temporal and spatial desynchronization. Additionally, we use an efficient multilevel filtering system accelerating the processes of fingerprint extraction and matching. This multilevel filtering system helps to rapidly identify potential similar video copies upon which the fingerprint process is carried out only, thus saving computational time. We tested with datasets of real copied videos, and the results show how our method outperforms state-of-the-art methods regarding detection scores. Furthermore, the granularity of our method makes it suitable for partial-copy detection; that is, by processing only short segments of 1 second length.

## Introduction

Nowadays, video-based applications flooded the Internet traffic. According to the Cisco Visual Networking Index [1], by 2019 it is projected that globally, Internet Protocol (IP) video traffic will be 80% of all IP traffic. Moreover, the sum of all forms of video (TV, video on demand (VoD), IP, and peer-to-peer (P2P)) will continue to be in the range of 80% to 90%. That means that every second, nearly a million minutes of video content will cross the network by 2019. In this scenario, methods for identification, indexing, searching and monitoring of video content should face the big challenge of manipulating such huge amount of videos in an efficient way.

Although current networking systems make efforts to deal with constraints related to real-time computing intensive applications such as mobile processing, cloud-based, and video decoding [2–7]; in video-based applications, there are additional challenges, especially the protection of copyrighted content.

The protection of copyrighted material is an important issue to address since piracy is a big problem in video distributions. The total cost of piracy is estimated close to US$20.5 billion each year [8]. Although there are many detection methods, security systems and even laws trying to fight piracy, piracy is still a profitable business; mainly encouraged by the ease of obtaining, uploading and downloading infringing content over peer-to-peer and other systems such as real-time streaming and distribution via cyberlockers [9]. Moreover, to pass unnoticeable through filters for copyrighted content, illegal videos are transformed or attacked by signal processing ranging from simple attacks to much more elaborate combinations of them. Some intentional video attacks are:

- The Decrease in quality and loss of information: compression, noise addition, contrast/brightness/gamma change, frame rate/bit rate change, frame drop/addition, etc.
- Post-production or edition: picture in picture (PiP), flip (horizontal mirroring), addition of patterns/subtitles
- Camcording

Although visual transformations are commonly used, also audio component could be easily and independently transformed; for example with temporal attacks, such as speed change, time/pitch shifting and loss of information.

The most popularly used transformations in real cases are the insertion of patterns, scale changes and camcording [10]. Camcording is the unauthorized recording of a feature film as it is being played on a theater screen or projection, and the resulting recording is known as a camcord [8]. The film industry identifies camcording as a major problem, and industry representatives estimate that camcords are responsible for at least 90% percent of the first available versions of illegally distributed new release films [8]. For those reasons, camcording and edition attacks are considered as the most common visual attacks.

To monetize illegal video distributions are required monitoring and copy detection systems. Video monitoring and copy detection systems are based mainly on passive content fingerprinting and watermarking [11, 12]. In contrast with watermarking, passive fingerprinting (also known as robust hashing or content-based copy detection) does not embed any information but it analyzes the content to determine their unique characteristics. The identified pattern or fingerprint of a reference video is stored in a database (offline processing) and can be used for recognizing the content in the future when a query video is required to be compared (online processing). In that way, a fingerprint represents a short, robust and distinctive content description allowing fast and privacy-preserving operations [13]. Despite many fingerprinting methods and commercial applications for video monitoring and copy detection have been proposed, these are not able to achieve the detection combining the desirable characteristics of a) robustness against the intentional attacks in real cases (mentioned above), b) effectiveness in the detection of short partial copies [10] and c) efficiency for real-time applications [14].

This paper presents a novel methodology to address the video copy detection challenge. For the latter, we focus on two main issues that current state-of-the-art methods do not address or simply struggle with: partial-video copy detection using short segments, and testing over real video copies rather than simulated transformations. Aiming at dealing with these issues, we propose using a set of visual fingerprints whose combination leverages the performance in the partial-video copy detection task, thus outperforming the results obtained by state-of-the-art methods. Additionally, to decrease the computational cost during online processing, we use a multilevel fingerprint extraction and matching process to gradually filter the most similar copies, which saves video frame processing. Moreover, our proposed method uses low memory footprint fingerprints, hence leading to a more efficient searching stage, suitable for large video databases.

The rest of this document is organized as follows: in section related work, a brief review from the state-of-the-art about fingerprinting methods is presented. In section proposed method are explained the core processes of our proposal that are video processing, keyframe generation, fingerprints extraction and multilevel matching processes. In section experimental results and discussion, the experimental setup, results, and their respective analysis are described. Finally, concluding remarks are drawn.

## Related work

In general, the extraction process of a passive fingerprint can be summarized in the following main steps: 1) downsampling the video signal, 2) feature extraction/description and 3) fingerprint compaction if necessary. The goal of the first step is to reduce the processing information decreasing the computational cost of the method. In video downsampling, the selection or generation of a frame that is representative of a video segment (*a.k.a* keyframe) is commonly used. Additionally, the frames (or keyframes) may be downsized and color space changed. There are many techniques for obtaining keyframes in the literature [15–18]. However, the generation of a keyframe represents an extra computational cost, for that reason most passive fingerprinting methods randomly sample frames as keyframes (*e.g.* 1 fps) [19, 20].

After video processing, the following steps are for the video fingerprint extraction, which can be generated from global, local, spatial or temporal features. Global features tend to be faster to compute and in a more compact way than local features, desirable characteristics for applications that require real-time processing. In counterpart, local features tend to be more robust against video transformations, whereas temporal features give extra information to persist against temporal desynchronization attacks.

For example, in [19], the color correlation histogram generates a global fingerprint. Although that method achieves better results against many attacks using long video segments (10s-200s), it is very susceptible to simple attacks that change the color correlation. Another method based on global features was presented in [21], where the Discrete Cosine Transform (DCT) coefficients obtained from temporary informative representative images (TIRI) are used as the fingerprint. In [22] is presented another method based on the combination of TIRI-DCT, brightness-sequence feature and a Hadoop platform (Hadoop is a distributed computing platform suitable for applications with a large data set). Although the specialization of this method is for a large volume of data and resistant against video processing transformations, fingerprints based on DCT coefficients are not robust against common content-changing attacks (*e.g.* in post-production).

The use of a Discrete Wavelet Transform (DWT) to generate a global fingerprint is proposed in [23]. That fingerprint is robust against luminance change, salt and pepper noise, Gaussian noise, text insertion, letter box, rotation, frame dropping, and time shifting.

In [24] two binary global descriptors (PHOG and GIST) are combined to generate a fingerprint. Using an additional pre-processing step to detect and revert the generated effects of some edition attacks, that method resists against cropping, flipping and PiP attacks. However, PHOG and GIST can not resist the insertion of patterns because it affects the global feature representation.

Other method which combines global features is presented in [25]. That method generates a video hash (ST-Hash) based on visual saliency, combining spatial and temporal hashes with an optimized weight coefficient. ST = Hash is based on TIRI and a visual hash (V-Hash) based on representative saliency maps (RSM). That combination outperforms the precision rate over the use of only ST-Hash proposed in [21]. A different combination of two kinds of global features is proposed in [26], in that the use of TIRI and a representative saliency map was evaluated with simulated signal processing attacks.

Like the global features, local keypoint detectors/descriptors are widely used due to their robustness and discriminability. However, the necessity of descriptor compaction induces the discriminability loss. In general, with a selected combination of feature extraction, fingerprinting methods tend to be more robust against video attacks while increasing the computational cost. For instance, in [20] four detectors are combined: SIFT, SURF, DCT for visual component and WASF for audio component. Other methods that combine visual and acoustic information are proposed in [27, 28]. In [29], the combination of robust local and global visual feature representations with a time-variant jitter synchronization gives robustness against scale, orientation and affine transforms. Most current fingerprinting methods are focused on achieving robustness against compression, random noise addition, resizing, rotation, cropping and/or frame rate changes [14]. Some methods are robust only against some specific attacks, for example, flip and rotation [30, 31]. Nevertheless, just a few methods are robust against camera recording or temporal domain changes [23, 29, 32], which are very common and severe attacks [10].

Moreover, in TREC Video Retrieval (TRECVID) workshop series, a content-based copy detection (CCD) task ran over a period of 4 years (2008 to 2011). The report of those competitions concludes that current state-of-the-art methods, which are robustness focused, are not suitable for real-time applications due to their high computational cost [33]. They also reported that current methods are not focused on applications where false alarms (NOFA apps) are not tolerated nor focused on generating compact fingerprints for improving their search in databases [14, 33–35]. Also those methods, which present near perfect scores under simulated attacks in TRECVID competition, present a low performance under non-simulated or real partial-video copies (VCDB benchmark) [10]. According to [10], the performance of current methods for partial copy detection is far from satisfactory.

## Proposed method

Fingerprinting methods are composed of two stages: the offline and the online processing. In the former, the fingerprints of reference videos are extracted and saved in a database. In the latter, a query video segment is matched or rejected. That is, the fingerprints of the query segment are extracted and searched in reference fingerprints database. As it is shown in Fig 1, a video processing, and keyframe generation steps are required previous to fingerprints extraction. Video processing, keyframe generation, fingerprints extraction and multilevel matching are described in next subsections. The optimization of indexing and searching processes is out the scope of this work.

**Fig 1:**
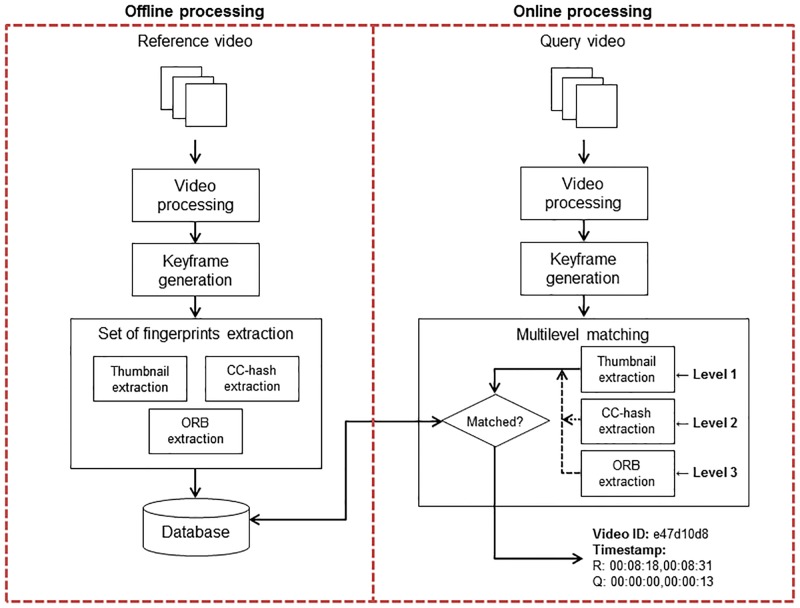
Proposed visual fingerprint extraction.

### Video processing and keyframe generation

Video processing has two aims: 1) to decrease the computational load and 2) to diminish the effects of video format changes of the transformed copies. With these aims, a video segment is downsampled to *n* frames per second (*n*_*fps*_), and then, the downsampled frames are resized to a fixed length of *M* × *N* pixels, where *N* = 2 × *M*. To cope with color format changes while we take advantage of the color correlation, we use two models, a transformation to RGB to extract its color correlation histogram as a fingerprint and the intensity of pixels (conversion to gray scale format) to extract the other two fingerprints. After video processing, a keyframe is generated per each video segment of length *l*_*s*_ seconds. The pixel values from all the frames in that segment are averaged into a single keyframe. That keyframe is first generated in RGB color model *Kf*_*RGB*_ and then its grayscale version is generated *Kf*_*temp*_.

A very common transformation in copy detection systems is the flip attack. Flip attack changes the position of pixels although not their values. Such localization loss makes most of the descriptors not robust against this transformation. In counterpart, the frame color relation does not change in a flip attack. Due to these facts, we use a frame folding simulation as a counter attack before fingerprints extraction in the gray scale keyframe version, similar to the presented in [30]. The folding frame is the average of the reflected right half of *Kf*_*temp*_ within its left half, generating a keyframe *Kf* of size *M* × *M*.

An important strategy to generate representative keyframes is to detect and merge similar sequential keyframes. To this fashion, after keyframe generation, we extract in advance its fingerprints and we compare them with the obtained fingerprints from the previous keyframe. In our proposed method we use a set of a local and two global fingerprints, whose extraction will be explained in next subsection. If the similarity between each pair of consecutive set of fingerprints is under an established threshold *θ*_*std*_, then those keyframes are different enough and their fingerprints and timestamps are saved. Otherwise, the strongest set of fingerprints is saved and the timestamp of those segments is updated. Due to local descriptors could be more discriminant under some transformations, the method keeps the fingerprints with a greater number of local features.

### Extraction of visual fingerprints

In large video databases, compact fingerprints are desired to optimize searching. As mentioned before, the robustness is also required to avoid false alarm cases. However, there is a tradeoff between robustness and fingerprints dimension. Additionally, in large databases as well as real-time applications, the low computational cost for fingerprints extraction is a concern. Taking into account that existing video transformations and their intensities are very diverse, all these desirable qualities are difficult to achieve. We propose to extract a set of independent fingerprints, each one selected for a specific purpose satisfying the desirable qualities exposed before. We use three fingerprints to describe a video segment: 1) a thumbnail, 2) the color correlation histogram and 3) ORB descriptors. The former two are global fingerprints of a generated keyframe while the last one refers to local features. The set of these fingerprints can be compared easily, due to the thumbnail is a small image, and the other two are binary fingerprints.

#### 1) Thumbnail

This representation is taken as a global fingerprint of the keyframe. The keyframe is downsized to (30 × 30) pixels; this small size is sufficient to represent the keyframe without fine details [36]. This is inferred from the way the human brain recognizes images. First, it uses low-level frequencies of an image to filter results and then high levels to refine detection [37, 38]. This global fingerprint can filter similar video copies and it is used for a first level filtering in the matching process.

#### 2) CC

Color Correlation (CC) is defined as the arrangement of red, green, and blue color components in order of intensity [19]. To extract a CC fingerprint, we follow the methodology proposed in [19]. The RGB keyframe is divided into 16 × 16 non-overlapped blocks, each block is then represented by the average intensities for the RGB components. For this sub-image we generate a normalized color correlation histogram. To this aim, it is necessary the evaluation of the following six cases:

1. *R*_*xy*_ > *G*_*xy*_ > *B*_*xy*_
2. *R*_*xy*_ > *B*_*xy*_ > *G*_*xy*_
3. *G*_*xy*_ > *R*_*xy*_ > *B*_*xy*_
4. *G*_*xy*_ > *B*_*xy*_ > *R*_*xy*_
5. *B*_*xy*_ > *R*_*xy*_ > *G*_*xy*_
6. *B*_*xy*_ > *G*_*xy*_ > *R*_*xy*_

Where, *R*_*xy*_, *G*_*xy*_ and *B*_*xy*_ represent the intensities of red, green and blue components of every pixel (*x*, *y*) in the keyframe. The resulting six real numbers are truncated, and the first five numbers are stored in a binary form as the feature for the keyframe. The resultant CC fingerprint for each keyframe is represented by only 35 bits. This color correlation histogram is a global feature without any spatial information about the keyframe, and it is robust mainly against scaling, rotation and flipping.

#### 3) ORB

It is a binary detector-descriptor proposed in [39]. It has a good performance at low cost and comparable precision/recall results to other widely used descriptors such as SURF and SIFT [40]. Each ORB binary feature is represented by 32 bytes. The algorithm uses FAST [41] in pyramids to detect stable keypoints, which is a rapid and efficient corner detection method. After keypoints detection, the algorithm selects the strongest features using FAST or alternative the Harris response, finding their orientation, and finally, computes the descriptors using BRIEF [42]. BRIEF is a binary descriptor robust against viewpoint changes, compression artifacts, illumination changes and image blurring [43]. This combination gives ORB descriptor a fast processing of visual features with a short representation. The extracted ORB features form the fingerprint of each keyframe.

### Multilevel matching process

In the offline processing, each video *v*_*i*_ of the reference dataset *RV* is processed and segmented in keyframes. For each keyframe *rKf*_*ij*_, where *i* is the reference video and *j* the corresponding segment number, a set of three fingerprints *rFP*_*ij*_ is extracted and stored: 1) *rTh* which refers to the global fingerprint based on thumbnail, 2) *rCC* which refers to the global fingerprint based on color correlation and 3) *rORB* which refers to the local fingerprint (ORB descriptors). Additionally, the timestamp related to keyframe segmentation is saved as the initial and final time for the segment. During online processing, for each query video *qv*_*k*_, the keyframe segmentation is performed with the same process than in offline processing. However, the set of fingerprints per keyframe is extracted by levels according to the filtering scheme presented in Algorithm 1.

**Algorithm 1** Multilevel matching process

 Per each *qv*_*k*_ find match in *rFP*_*ij*_

1: **Level 1: extract *qTh***

2: *C* = *correlation*(*qTh*, *rTh*)

3: **if** *C* > = *θ*_*Th*_ **then**

4:  **Level 2: extract *qCC***

5:  **if** There is *qCC* and *rCC* **then**

6:   *D* = 1 − *distance*(*qCC*, *rCC*)

7:   **if** *D* > = *theta*_*CC*_ **then**

8:    **Level 3: extract *qORB***

9:    **if** There is *qORB* and *rORB* **then**

10:     *E* = *match*(*qORB*, *rORB*)

11:     **if** *E* > = *theta*_*ORB*_ **then**

12:      save *ID*, *timestamp* of *rFP*_*ij*_

13:      *SimML* = *w*_Th_*C + *w*_CC_*D + *w*_ORB_*E

14:     **end if**

15:    **else**

16:     save *ID*, *timestamp* of *rFP*_*ij*_

17:     *SimML* = *w*_Th_*C + *w*_CC_*D

18:    **end if**

19:   **end if**

20:  **else**

21:   **if** There is *qORB* and *rORB* **then**

22:    *E* = *match*(*qORB*, *rORB*)

23:    **if** *E* > = *theta*_*ORB*_ **then**

24:     save *ID*, *timestamp* of *rFP*_*ij*_

25:     *SimML* = *w*_Th_*C + *w*_ORB_*E

26:    **end if**

27:   **end if**

28:  **end if**

29: **end if**

 Return highest *SimML*, *ID*, *Timestamp* for *qv*_*k*_

#### Evaluation of distance and similarity score

In Algorithm 1, *correlation*(*qTh*, *rTh*) refers to the Normalized Crossed Correlation (NCC) between the query and reference thumbnail pair. The NCC coefficient is given by Eq (1), where $\bar{qTh}$ and $\bar{rTh}$ are the mean values of keyframes *qTh* and *rTh* respectively.

$$\begin{array}{c} C=\frac{\sum_{m}\sum_{n}\left(qTh_{mn}-\bar{qTh}\right)\left(rTh_{mn}-\bar{rTh}\right)}{\sqrt{{(\sum_{m}\sum_{n}\left(qTh_{mn}-\bar{qTh}\right)}^{2}){(\sum_{m}\sum_{n}\left(rTh_{mn}-\bar{rTh}\right)}^{2})}} \end{array}$$ (1)

To compare a pair of CC binary fingerprints we use the Hamming distance, that is *distance* = *mean*(*xor*(*qCC*, *rCC*)). The same distance metric is used for a pair of ORB fingerprints (*qORBrORB*). The value *E* in algorithm 1 refers to the percentage of total ORB features matched per keyframe, being 1 a perfect score for identical keyframes. To increment the value of E, we define the distance *E* = 1 if 70% to 100% of the keypoints matched, *E* = 0.9 if 40% to 69% of the keypoints matched and *E* = 0.8 and *E* = 0.7 for the 20%-39% and 1%-19% of keypoints matched, respectively; if there were not matched keypoints then *E* = 0.

Due to during matching process each pair of fingerprints is evaluated separately, the total similarity score per query *SimML* corresponds to the weighted average of individual distances. The assigned weights are *w*_*Th*_, *w*_*CC*_, and *w*_*ORB*_ respectively.

## Experimental results and discussion

In the following subsections, the experimental setup is defined, the video datasets and video transformations used in the experimental process are presented, and a discussion about the obtained results is given.

### Experimental setup

Experiments were carried out in a PC with Intel i7 processor at 3.4GHz and 16GB in RAM using MATLAB *R*2015*b*. Additionally, we employed MEXOpenCV [44] which links the ORB author’s implementation in OpenCV 2.3 to MATLAB.

#### Parameters setting

The parameters in video processing were empirically selected. For video downsampling, different rates were used (30, 20, 10, 5 and 1 fps), and, then we generated a keyframe from different video segment lengths (1, 2, 4, 6, 8, and 10 seconds length). These segments dimensions are based on a standard client buffer length for streaming applications, which is about 6 to 10 seconds [45]. Additionally, we used shorter segments in order to assess the precision of our method with short copied videos. In the experiments carried out, good results in terms of execution time and precision were obtained using a downsampling rate of *n*_*fps*_ = 5 fps with a short segment of *l*_*s*_ = 1 second length. In many related works, it is common to downsize each frame to a QCIF standard size (*e.g.* [46, 47]). However, using a bigger keyframe (*e.g.* *M* × *N* = 320 × 640 pixels) it is possible to extract more local features achieving better detection scores. In the matching process the corresponding thresholds were: *θ*_*std*_ = 0.65, *θ*_*Th*_ = 0.50, *θ*_*CC*_ = 0.75, *θ*_*ORB*_ = 0.70. In the first level step a minor threshold is enough to select similar reference videos, whereas the subsequent filtering steps are more judicious. We use adaptive similarity weights per level in the matching process. Those weights are presented in Table 1. A video detection is declared if *Sim*_*ML*_ ≥ *θ*_*ML*_ and *θ*_*ML*_ = 0.65.

**Table 1:** Fingerprint weights.

	Th		CC		ORB	
Level	*C*_*m*_	*w*_*Th*_	*D*_*m*_	*w*_*CC*_	*E*_*m*_	*w*_*ORB*_
*L*_*CDE*_	1	0.25	1	0.25	1	0.50
*L*_*CDE*1_	1	0.40	1	0.50	−1	-
*L*_*CD*_	1	0.40	1	0.60	0	-
*L*_*CE*_	1	0.40	0	-	1	0.60

In this table are presented the selected weights *w*_*Th*_, *w*_*CC*_ and *w*_*ORB*_, for each fingerprint set according to the matching level. In columns *C*_*m*_, *D*_*m*_ and *E*_*m*_ the match results are indicated, where a value of 1 indicates fingerprint matched, 0 means fingerprint not found and −1 means fingerprint not matched.

### Datasets

For our evaluation we used two public video datasets: VCDB [10] and ReTRiEVED [48]. The ReTRiEVED dataset was designed to assess the quality of service in video transmissions and for this, a number of attacks are simulated in order to evaluate their effects on perceived quality, however, such benchmark can also be used to assess fingerprinting methods and their robustness, as we will describe in more detail in the following sections.

The VCDB dataset was specially intended to test partial copy detection in real videos, whose attacks were induced by external users to those who collected the dataset, which offers the opportunity to assess fingerprinting methods against non-controlled attacks.

Inspired by the experimental setup described in [10] and aiming to carry out a thorough evaluation of our method, we generate three different pools of videos. One of them, using the reference videos and their transformed versions from the ReTRiEVED dataset. The second one, using the same reference videos from the ReTRiEVED dataset transformed with additional attacks presented in the next subsection. Finally, we constructed a pool of videos for each query and its corresponding videos containing the partial copies (copies of the query) from the VCDB dataset. Every video in each pool was segmented into query segments of 1-second. Then, for each pool, we carried out a cross-fold-like validation scheme where we left 1 video out from the pool to be used as a reference video, while the rest are used as video queries for which the fingerprinting methods should detect that the query contains a partial video copy of the reference video, once this is done for all the videos in the pool, the reference video is put back into the pool and then a different video, in the pool, is chosen as the new reference video and left out of the pool to repeat this procedure. This process is repeated until all the videos in the pool have been used as reference videos.

### Video attacks

As a reference, the transmission attacks and parameters elaborated in the ReTRiEVED dataset are presented in Table 2 (see [48]). In addition to those transmission attacks, we transformed each reference video using the combination of attacks presented in Table 3. Using this set of attacks, we assessed our method against commonly simulated attacks.

**Table 2:** Attacks parameters in ReTRiEVED dataset.

Attack ID	Attack	Parameters						
D	Delay (ms)	100	300	500	800	1000	-	-
J	Jitter (ms)	1	2	3	4	5	-	-
PLR	Packet Loss Rate (%)	0.1	0.4	1	3	5	8	10
R	Throughput (Mbps)	0.5	1	2	3	5	-	-

In this table are presented the parameters of video attacks in ReTRiEVED dataset which are considered acceptable parameters of Quality of Service (QoS) based on ITU recommendations, opinion models for video telephony applications and ETSI recommendation on speech and multimedia transmission quality [48].

**Table 3:** Additional attacks on ReTRiEVED video dataset.

Attack ID	Attack	Parameters
FLIP	Flip	Horizontal flip
DQ1	Frame rate change	20 fps
	Contrast change	+10%
	Noise Addition	Gaussian: mean = 0; variance = 0.01
DQ2	Frame rate change	20 fps
	Brightness change	+10%
	Cropping	10% of the frame border
DQ3	Frame dropping	10% dropped
	Rotation	+5^*o*^
	Brightness change	+10%
FLIP + DQ2	Flip	Horizontal flip
	Frame rate change	20 fps
	Brightness change	+10%
	Cropping	10% of the frame border
PROJ	Projection	*θ* = 1^*o*^ using MATLAB projective2d = ([cos(*θ*) -sin(*θ*) 0.001; sin(*θ*) cos(*θ*) 0.001; 0 0 1]);
PROJ + DQ2	Projection	*θ* = 1^*o*^ using MATLAB projective2d = ([cos(*θ*) -sin(*θ*) 0.001; sin(*θ*) cos(*θ*) 0.001; 0 0 1]);
	Frame rate change	20 fps
	Brightness change	+10%
	Cropping	10% of the frame border
POST1	Insertion of patterns	Standard image baboon.jpg 30x30 pixels
POST2	Subtitles	35 characters are inserted [49] in the first frame of each second of the video
POST3	PiP	Original video resized to 90% at front
POST4	PiP	Original video resized to 90% at front
	Insertion of patterns	Standard image baboon.jpg 30x30 pixels
	Subtitles	35 characters are inserted [49] in the first frame of each second of the video

Common simulated visual transformations used in state-of-the-art experimental results [14] and combinations of them.

It is important to highlight that the percentages of found attacks in real copies are different from those in simulated datasets. According to [10], insertion of patterns is a widely used attack while PiP is not a popular transformation in real cases. Hence, for the evaluation of our method in real cases, we used the VCDB dataset which contains real partial copies of selected videos rather than synthetic attacks. The transformations included in VCDB dataset are: insertion of patterns (36% of videos in dataset), scale changes (27%), camcording (18%) and PiP (2%).

### Evaluation metrics

F-score is widely used to evaluate copy detection tasks. F_1_-score is the balance harmonic mean between precision (PPV: Positive Predicted Value) and recall (TPR:True Positive Rate). These parameters are given by Eq (2), where *TP*, *TN*, *FP*, *FN* are True Positive, True Negative, False Positive and False Negative values respectively, and *P* = *TP* + *TN*. Results with F-score tending to 1 are preferable due to effective copy detection methods should avoid false alarm errors.

$$\begin{array}{c} F_{\beta }-score=\frac{(\beta^{2}+1)PPV*TPR}{\beta^{2}PPV+TPR} \end{array}$$ (2)

$$\begin{array}{c} TPR=\frac{TP}{P} \end{array}$$ (3)

$$\begin{array}{c} PPV=\frac{TP}{TP+FP} \end{array}$$ (4)

### Evaluation using ReTRiEVED dataset

This dataset contains 184 test videos obtained from eight source videos of different content, characterized by spatial and temporal information and different motions. These videos simulate possible artifacts during transmission; including jitter, delay, Packet Loss Rate (PLR) and throughput. These transmission attacks have a considerable effect on perceived quality [48], which makes them suitable to test the robustness of any fingerprinting method.

In order to evaluate the detection scores of our proposed method, we compare the robustness of the extracted fingerprints (Th + CC + ORB) against other similar fingerprints: CST-SURF which is based on a compact representation of local keypoints, ST&V that combines visual and temporal compact features, and CC to test the robustness of this employed fingerprint against real or non-simulated attacks. The selected fingerprints from the state-of-the-art are briefly described in the following list.

- CC [19]. As mentioned in the proposed method section, in color correlation method each keyframe is divided into non-overlapping blocks. For each block, the red, green, and blue color components (RGB) are sorted according to their average intensities and the percentage of the color correlation is employed to generate a frame feature with a small size. Color correlation is robust against most typical video content-preserving operations, it is mainly robust against scaling, flipping, and rotation.
- ST&V Hash [25]. In this method the combination of two hashes is presented: 1) spatiotemporal hash (ST), which is based on TIRI (Temporal Informative Representative Images) and the intensity difference between adjacent TIRI blocks and 2) visual hash (V) generated according to the visual saliency distribution. ST&V hash is the XOR operation between ST and V hashes. This fingerprint is robust against some temporal attacks (frame dropping, frame rate conversion, and frame exchanging) and signal processing attacks (Gaussian noising, median filtering, histogram equalization, among others).
- CST-SURF [27]. CST-SURF is part of a spatiotemporal registration framework. CST-SURF is a compact representation of the mean of differences between *k* × *k* region-wise count of SURF interest points of consecutive frames. This compact form of SURF is designed to efficiently characterize the spatiotemporal content of frames while mitigating the issue of the high dimensionality of SURF features and the excessive computational load to compare them. CST-SURF is promising robust against camcording, edition, and signal processing attacks.

The obtained F_1_-scores of the tested different methods under simulated attacks are presented in Tables 4 to 8.

**Table 4:** F_1_-scores for delay attacks.

	Delay (ms)				
	100	300	500	800	1000
CST-SURF	0.5263	0.3283	0.3425	0.3384	0.3333
ST&V Hash	0.9818	0.9629	0.9642	0.9306	0.9622
CC	1	0.9818	0.9734	0.9615	0.9719
Th + CC + ORB (our proposed method)	0.9931	0.9933	0.9930	0.9929	0.9931

**Table 5:** F_1_-scores for PLR attacks.

	Packet Loss Rate (PLR%)						
	0.1	0.4	1	3	5	8	10
CST-SURF	0.4383	0.1587	0.3283	0.4657	0.3188	0.1311	0.1818
ST&V Hash	0.9345	0.9259	0.9230	0.9818	0.9158	0.9038	0.8785
CC	0.9821	0.9824	0.9909	0.9724	0.9824	0.9541	0.9473
Th + CC + ORB (our proposed method)	0.9929	0.9861	0.9930	1	1	0.9931	0.9932

**Table 6:** F_1_-scores for jitter attack.

	Jitter (ms)				
	1	2	3	4	5
CST-SURF	0.5066	0.1791	0.0563	0.0563	0.0540
ST&V Hash	0.9818	0.9391	0.8688	0.8205	0.8000
CC	0.9532	0.9009	0.8960	0.8870	0.8292
Th + CC + ORB (our proposed method)	0.9929	0.9932	0.9801	0.9664	0.9655

**Table 7:** F_1_-scores under throughput attack.

	R(Mbps)				
	0.5	1	2	3	5
CST-SURF	0.0895	0.3142	0.3188	0.3380	0
ST&V Hash	0.8965	0.9557	0.9642	0.9557	0.5915
CC	0.9593	0.9914	0.9913	0.9914	0.7341
Th + CC + ORB (our proposed method)	1	1	1	1	0.7475

**Table 8:** F_1_ detection scores for different fingerprints under common simulated attacks.

Attack ID	CST-SURF	ST&V Hash	CC	Th + CC + ORB (our proposed method)
None	1	1	1	1
FLIP	0.2727	0.3714	1	1
DQ1	0.0740	0.9052	0.9278	1
DQ2	0.2909	0.8536	0.9069	0.9911
DQ3	0.2173	0.7941	0.9350	0.9916
FLIP + DQ2	0.0377	0.2666	0.9494	1
PROJ	0	0.5714	0.9655	0.7407
PROJ + DQ2	0.1290	0.5853	0.7659	0.8367
POST1	1	0.9914	1	1
POST2	0.9142	0.9821	1	1
POST3	0.1714	0.6086	0.7450	0.7890
POST4	0.1379	0.7142	0.6153	0.7865

Transmission artifacts simulated in ReTRiEVED dataset have a considerable effect on perceived quality, due to this fact the detection of video copies based on content fingerprints is easily affected. However, due to the combination of different fingerprints in our proposed method, it can achieve high F_1_-scores for the detection of short video segments (1 second length). Particularly, when videos are subject to typical transmission errors such as delay, PLR, jitter and throughput (see Tables 4–7), our method achieves comparable to better F_1_-scores than the other tested methods. Due to loss of information and temporal desynchronization, the fingerprint based on CST-SURF presented the worst detection scores. For the other three fingerprints, we perform the extraction from the same generated keyframe, which has a better performance even under these kinds of attacks than frame by frame processing (such as in CST-SURF). The results show better detection scores when we use the Th + CC + ORB fingerprints combination than using a unique fingerprint (CC) or than other visual fingerprint combination (SV&V).

On the other hand, Table 8 presents the experimental results using a simulated combination of attacks, which includes the decrease in quality and edition. As it can be seen from Table 8, the combination of Th + CC + ORB outperforms detection in comparison with other fingerprints. An exception is when videos were attacked with projective transformations, in this particular case, even though CC has better matches, Th and ORB fingerprints introduce noise in the multilevel matching. This is mainly due to the folded frame in the video processing step. The simulated change of view greatly modifies the keyframe thumbnail in comparison with reference video and moreover, many ORB keypoints are lost. An interesting result is when PROJ attack is combined with DQ2, in this case, the additional processing (brightness change and cropping) counteracts the projective transformation severity. This example shows that in some cases a video processing could counteract some specific transformations. Another observation is that in real applications, video attacks not necessarily are similar to combinations of attacks employed in simulations [10].

Some remarks regarding the fingerprints combination of our proposed method are that the global fingerprint *Th* prevails especially under decrease in quality attacks. However, this kind of fingerprint has the disadvantage that by itself, is not enough discriminative in near-duplicate videos. This is mainly because this fingerprint does not have the finer details from each keyframe. For this reason, we use this fingerprint as an initial filter to single out similar videos in the multilevel system. On the other hand, the main advantage of the *CC* fingerprint is that color correlation histograms prevail against many attacks whereas the computational cost to compare this kind of fingerprint in the matching process is very low (the Hamming distance of 35-bit strings). Due to this reason, *CC* is used as a second filter in the multilevel system. In counterpart, the *ORB* fingerprint has better results under post-production transformations due to it is based on local keypoints. Hence, the combination of these fingerprints leverages the individual performance of the fingerprint methods. It is important to highlight that our proposed method has satisfactory detection scores even without the use of additional transformation detection and counterattack processes. However, as it was expected, under extreme decrease in quality transformations such as camcording, the proposed method presented the lower detection scores.

### Evaluation using VCDB dataset

**Fig 2:**
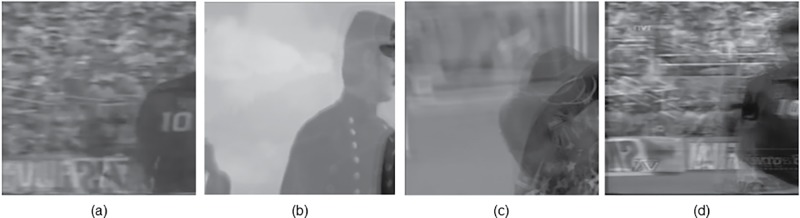
Example of false positive alarm. The figures correspond to matched keyframes of different videos in VCDB benchmark [10]: (a) Query video segment (baggio_penalty_1994), (b) (the_last_samurai_last_battle) correlation = 0.889; (c) (endless_love) correlation = 0.776; (d) corresponding keyframe of original video (baggio_penalty_1994) correlation = 0.674.

We additionally used VCDB dataset [10] as it offers a set of videos that were collected from the internet. These videos exhibit several attacks that were not controlled or synthetically induced by the authors of the dataset, which offers a great opportunity to assess the effectiveness of video copy detection algorithms against real attacks rather than simulated. The videos in this dataset have been affected by different attacks, some of which include insertion of patterns, camcording and scale change. The dataset consists of a collection of 28 queries and around 20 videos per query representing real partial copies. This dataset includes topics such as commercials, movies, music videos, public speeches, sports, etc. In total, the benchmark has 528 videos (approximately 27 hours) in the core dataset. In the evaluation presented in [10], the best recall rate on VCDB is close to 0.60 when the precision significantly drops to 0.20. In a similar evaluation, our method obtained a recall or *TPR* = 0.9294 and precision or *PPV* = 0.5052, which greatly outperforms those results reported in [10] that are recall = 0.6 and precision = 0.2. Regarding these results, we should indicate that the fingerprint based on the ORB descriptor is responsible for most of the false alarms in the video detection. The latter is due to the fact that ORB descriptors are not robust against strong changes in scale or loss of contrast, typically induced in camcording attacks. Another issue to deal with is the right balance between the weights and thresholds selected in matching process, because these parameters are essential for detection without false alarms. An example of a false positive result is showed in Fig 2, where ORB features were lost and the different keyframes have better matches with other videos rather than the original.

### Execution time and fingerprints dimension

In Table 9 the execution times for the fingerprints extraction of our method and other state-of-the-art methods are presented. These values include video processing and fingerprints extraction times in similar evaluation conditions for all the presented methods. In the case of our proposed method (Th + CC + ORB fingerprints based), the execution time is divided as follows: video processing = 0.0380*sec*, extraction of Th = 0.0009, extraction of CC = 0.0017 and extraction of ORB = 0.0034. On average it takes only 0.0440 seconds to process a video segment of 1 second length. Although color correlation is the fastest method, Th + DCT + ORB fingerprints (our proposed method) are more robust against the reported attacks.

**Table 9:** Execution times for different fingerprint extraction types.

Method	Ex.Time (seconds)
CC [19]	0.0204
ST&V Hash [25]	0.4373
CST-SURF [27]	0.4898
Th + CC + ORB(our proposed method)	0.0440

The dimension of the set of extracted fingerprints is as follows:

- Th: is represented by an small image of size 30 × 30 pixels per keyframe.
- CC: is formed by 35 bits per keyframe.
- ORB: this binary detector generates features of 32 bytes each one.

In summary, the dimensionality of our proposed method depends on the video content information, the changes in video scenes (extracted keyframes) and duration. Th and CC fingerprints have a fixed length per keyframe while ORB fingerprint generates 150 features per keyframe of 320 × 320 pixels on average. Nevertheless, binary fingerprints are faster for searching and matching purposes.

## Conclusions

We have presented an efficient content fingerprinting method based on the extraction of a set of independent binary global and local fingerprints. The combination of robust fingerprints gives to our method the capability to detect videos severely transformed. Our method aims at achieving robustness against the decrease in quality, edition, and temporal desynchronization attacks which are common in video transmissions. Furthermore, we have presented an evaluation of our method by testing it with real video copies rather than simulations using synthetic and controlled attacks, achieving better precision and recall results than the related works. Moreover, our method can detect short video segments of 1-second length, which is suitable for partial-copy detection.

We have also explored the use of light-weight global and local binary descriptors to achieve computational efficiency. In this sense, we propose to combine a global binary fingerprint based on color correlation with a local fingerprint based on the ORB binary descriptor, which brings the benefit of having a low computational cost in the matching stage.

On top of this, we propose to use a multilevel matching system that aims at identifying potential similar video copies upon which the binary fingerprints will be extracted, thus avoiding the processing of all the video queries. This multilevel system is based on a filtering stage (using a fingerprint based on the keyframe thumbnail) and a refinement stage (binary fingerprints). According to the performed experiments, using this multilevel system the operations (fingerprints extraction and similarity calculations) are reduced to one-fourth in comparison to extracting the full fingerprints set in a brute force search. Furthermore, the searching process can be improved using indexing and/or classification techniques.

Our experimental results show that, our method obtains better detection scores than the state-of-the-art methods tested in this work, for all simulated attacks excepting projective transformations, with lower computational time, and processing only short video query segments with a length of 1-second, which is suitable for partial video copy detection. Moreover, this short segment is processed at a speed of 0.0440 seconds in average, independently from the query video frame rate. Regarding the experiments with non-simulated attacks, our method obtains better precision and recall scores than those obtained with other methods in the literature.

### Future work

We have observed that in the case of video copy detection under non-simulated attacks, the local fingerprints lose effectiveness due to the aggressive global transformations suffered by the video frames. This is expected since there is a limitation of robustness for the ORB descriptor against changes in scale, image distortion and illumination changes. In order to address this, the use of different binary fingerprints more suitable for these kinds of attacks could be investigated.
